# Genetic and Epigenetic Determinants in Autoinflammatory Diseases

**DOI:** 10.3389/fimmu.2017.00318

**Published:** 2017-03-22

**Authors:** Damiana Álvarez-Errico, Roser Vento-Tormo, Esteban Ballestar

**Affiliations:** ^1^Chromatin and Disease Group, Cancer Epigenetics and Biology Programme (PEBC), Bellvitge Biomedical Research Institute (IDIBELL), Barcelona, Spain

**Keywords:** autoinflammatory diseases, epigenetics, DNA methylation, non-genetic factors, cryopyrin-associated periodic syndromes, Familial Mediterranean Fever

## Abstract

The concept of autoinflammation has evolved over the past 20 years, beginning with the discovery that mutations in the Mediterranean Fever (*MEFV*) gene were causative of Familial Mediterranean Fever. Currently, autoinflammatory diseases comprise a wide range of disorders with the common features of recurrent fever attacks, prevalence of hyperreactive innate immune cells, and signs of inflammation that can be systemic or organ specific in the absence of pathogenic infection of autoimmunity. Innate immune cells from the myeloid compartment are the main effectors of uncontrolled inflammation that is caused in great extent by the overproduction of inflammatory cytokines such as IL-1β and IL-18. Defects in several signaling pathways that control innate immune defense, particularly the hyperreactivity of one or more inflammasomes, are at the core of pathologic autoinflammatory phenotypes. Although many of the autoinflammatory syndromes are known to be monogenic, some of them are genetically complex and are impacted by environmental factors. Recently, epigenetic dysregulation has surfaced as an additional contributor to pathogenesis. In the present review, we discuss data that are currently available to describe the contribution of epigenetic mechanisms in autoinflammatory diseases.

## Introduction

Autoinflammatory diseases are a growing group of debilitating and chronic conditions characterized by overt inflammation that is often systemic and manifests as recurrent fever episodes. Hyperreactive innate immune cells contribute largely to the pathogenesis of these diseases, and patients who display this pathology correlates with increased levels of acute-phase proteins and inflammatory cytokines in the plasma. Originally, the term autoinflammation was coined to describe the occurrence of apparently unprovoked episodes of inflammation in the absence of self-reactive T cells and/or high titers of autoantibodies, as well as in the absence of any detectable infectious agent (1). Many of the autoinflammatory syndromes display systemic and/or organ-specific inflammatory features such as recurrent and episodic periodic fever, serositis, arthritis and/or cutaneous inflammation, overproduction of IL-1β, and activation of innate immune cells, particularly monocytes (2). Albeit initially the term autoinflammatory diseases only applied to those prototypical hereditary monogenic periodic fever syndromes, such as cryopyrin-associated periodic syndromes (CAPS) and Familial Mediterranean Fever (FMF), the list has now expanded as a consequence of the application of emerging technologies, such as next-generation sequencing, and comprises an increasing number of newly described monogenic disorders caused by mutations of inflammation-related genes. There is also increasing evidence that epigenetic dysregulation participates in the pathogenesis of these diseases [Table 1; reviewed by Stoffels and Kastner (3)]. In addition, autoinflammatory diseases also include a few multifactorial and complex diseases, such as Behçet’s disease and Crohn’s disease (CD), which not only involve the participation of multiple alleles but also a number of environmental factors (2, 4). Also, it is now accepted that there is a continuum of disorders in the inflammatory spectrum that ranges from autoimmune diseases at one end to autoinflammatory at the other, with several mixed complex conditions that display both features of innate and adaptive immune dysregulation (5, 6). This growing spectrum of conditions indicates the existence of a highly complex etiology and pathophysiology of inflammatory diseases, even in the case of monogenic diseases, where additional agents, besides the causative gain-of-function mutations, may have a relevant impact on the clinical course of the disease. In autoinflammatory diseases, a failure in the regulation of the defense mechanisms of innate immune cells, which responds to pathogen-expressed molecules or molecules signaling cellular stress, and the orchestration of a response to such insults with the production of proinflammatory cytokines such as IL-1β or IL-18 (2) are central to pathology. Genetic inheritance in autoinflammatory disorders varies depending on the specific disease and has been a subject of controversy. FMF is mostly transmitted in an autosomal recessive manner, which requires mutations in both alleles of the Mediterranean Fever (*MEFV*) locus, encoding the sensor protein pyrin that is expressed in neutrophils, eosinophils, and cytokine-activated monocytes (7). Interestingly, there have been reports of several cases of FMF patients that are heterozygous for the *MEFV* allele, with only one allele displaying a mutation or, in even rarer cases, no detectable mutation, and yet still associate with the development of disease (8–10). Several groups studying the FMF phenotype in *MEFV* mutation-negative patients found the phenotype to be milder, with a late disease onset and a lower rate of familiar history of FMF. However, the unequivocal existence of such mutation-free patients suggests the existence of additional causes for disease development including mutation in alternative genes, and perhaps the occurrence of epigenetic dysregulation. Identification of those alterations is essential for patient diagnosis and management.

**Table 1:** **Autoinflammatory disorders and evidence of epigenetic contribution to pathogeny**.

Mutated gene	Disease	Effector cytokine	Data on epigenetic regulation
**Hereditary monogenic periodic fever syndromes**			
*MEFV*	Familial Mediterranean Fever	IL-1β	Yes (38)
*TNFRSF1A*	TRAPS	IL-1β	No
*MVK*	Hyper IgD syndrome	IL-1β	No
*NLRP3*	Cryopyrin-associated periodic syndromes [familial cold autoinflammatory syndrome (FCAS), Muckle–Wells syndrome, neonatal-onset multisystem inflammatory disease/CINCA]	IL-1β	Yes (40)
*NLRC4*	NLRC4-MAS	IL-1β/IL-18	No
*PSTPIP1*	PAPA	IL-1β	No
*NLRP12*	FCAS2	IL-1β	No
**Antagonist deficiencies**			
*IL1RN*	DIRA	IL-1β	No
*IL36RN*	DITRA	IL-36	No
**Complex autoinflammatory disorder**			
	Behçet	IL-6/IL-1β	Yes (41, 48, 49)
	CRO/chronic recurrent multifocal osteomyelitis	IL-10/IL-1β	Yes (42, 43)
	Crohn	IL-19/IL-3/IL-27	Yes (50, 51)

In the case of CAPS, inherited dominant autosomal gain-of-function mutations of NOD-like receptor, *NLRP3* gene encoding cryopyrin, are responsible for the overactivation of the inflammasome (11–13). In fact, CAPS is a spectrum disorder that includes, in increasing order of severity, the familial cold autoinflammatory syndrome (FCAS), Muckle–Wells syndrome, and neonatal-onset multisystem inflammatory disease (NOMID), caused by sporadic *de novo* mutations in the same gene (otherwise termed chronic infantile neurologic cutaneous and articular syndrome/CINCA) (14). Several lines of evidence, including the existence of mutations with different degrees of penetrance leading to a gradient of disease severity and heterogeneous phenotypes in terms of disease progression that arises from identical germline mutations, suggest that additional factors contribute to pathophysiology of hyperinflammation. Moreover, a great number of cases (as much as 40% in the case of NOMID/CINCA for conventional sequencing) are considered “genetic orphans,” i.e., patients without any identified associated mutations, which further supports this notion. In some of these cases, the existence of mosaicism restricted to the myeloid compartment has been reported; however, there is the possibility that, in some cases, non-genetic mechanisms could lead to autoinflammation. For example, it is plausible that, in addition to the occurrence of specific mutations, certain amplification loops establish vicious circles that increase IL-1β production and inflammation. The complexity of genome regulation in autoinflammatory diseases is reflected in CAPS, where it is extensively agreed that the lack of genetic confirmation for some patients does not exclude their diagnosis (15). In the cases of complex autoinflammatory disorders where heritability models are not well established, it is entirely possible that, although there may be a genetic component that contributes to certain parts of disease, both genetic and environmental/epigenetic factors may define pathogenicity, and this applies to disorders like Behçet’s disease, inflammatory bowel disease (IBD), and chronic recurrent multifocal osteomyelitis (CRMO) among others. It has been long recognized that environmental factors contribute to the establishment of pathological immune responses as well as the development and severity of inflammatory immune disorders, and twin studies have been valuable to determine the extent of genetic and non-genetic contributions, such as in the case of IBD (16). Since epigenetic mechanisms establish a diversity of links between the environment and the regulation of the genome, understanding epigenetic control within the innate immune compartment is crucial to fully grasp the etiology of autoinflammatory disorders.

## Control of Innate Immune Cell Function

**Figure 1:**
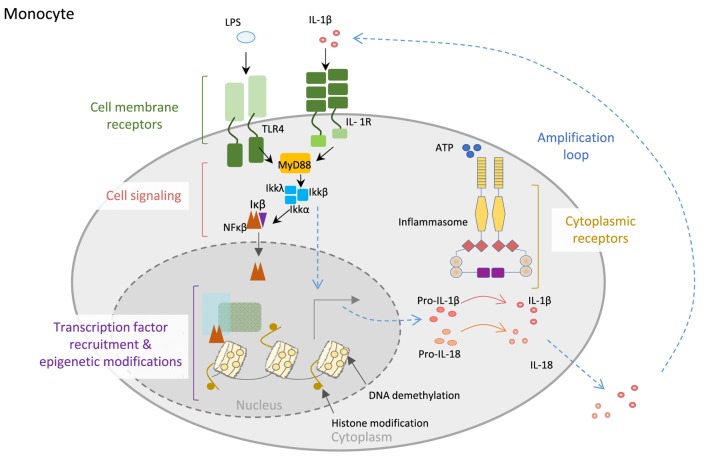
**Control of innate immune cell function**. Monocyte cell membrane receptors as TLR4 and IL-1R allow communication between the cell and the environment. Engagement of the receptors by their ligands (LPS or IL-1β) triggers cell signaling cascades, allowing transcription factors, particularly NF-κB translocation into the nucleus, where it recognizes specific regions of the DNA and recruits other transcription factors, as well as epigenetic enzymes, like TET2 (protein involved in DNA demethylation in myeloid cells). Both the binding of transcription factors to the DNA and the epigenetic modifications of the DNA will increase the expression of inflammatory genes, like the inflammasome complex components. Posttranscriptional modifications of inflammasome proteins play a crucial role in the formation of the inflammasome complex, leading to the activation of caspase-1, which then is able to process the proinflammatory cytokines IL1-β and IL-18 into mature bioactive IL-1β and IL-18 cytokines that are secreted to the external media, creating an inflammatory microenvironment. Importantly, IL-1β is able to amplify its own signal through the binding to IL-1R.

The acquisition of full host protection requires proper orchestration and balance between resistance and tolerance, with the former being necessary to maximally reduce pathogen burden and the latter to minimize self-tissue damage by inflammation (17). Innate immune cells, including monocytes, macrophages, and neutrophils, are in the first line of defense and hence are equipped with very specialized molecular machinery aimed at sensing and destroying invading pathogens and restoring homeostasis. Pattern-recognition receptors that recognize pathogen-associated molecular patterns, and non-microbial stress signals, known as danger-associated molecular patterns, constitute the sensors that trigger upon recognition of their substrates during an inflammatory response. Many of these receptors, including the toll-like receptors and C-type lectin receptors, are located on the cell membrane in contact with the extracellular milieu, whereas others are cytoplasmic, such as the inflammasome-participating NOD (nucleotide-binding oligomerization domain)-like receptors and AIM2 (absent in melanoma) family of receptors (18). Inflammasomes are a key component of such defensive machinery that consist of multimeric cytoplasmic platforms that ensemble upon recognition of an insult and respond by activating pro-caspase-1, leading to proteolytic processing and release of IL-1β and IL-18, and pyroptosis (Figure 1) (19, 20).

Innate immune cells, in particular monocytes and macrophages, rely on epigenetically controlled functional reprogramming in order to coordinate a proper response once stimuli are detected. During the differentiation of monocytes to macrophages, whole transcriptome and epigenome studies have shown that substantial changes affect ~19 Mbp, which is equivalent to 0.6% of the human genome. Epigenetic changes affect the activity of promoters (H3K4me3/H3K27ac) and distal regulatory elements that are presumed enhancers (H3K4me1/H3K27ac) to a similar extent (21).

The acquisition of a trained or tolerant state in macrophages upon encounter with an external stimulus of microbial origin is associated with changes in around 0.12% of the entire monocyte/macrophage epigenome. There is also around 12% difference in the expression of transcription factors, which dictates the specific antimicrobial response, which also results in an immunological memory coded in the chromatin that will have an impact on how the cell reacts to future challenges. Furthermore, transcriptomic analysis of the acquisition of functional memory by macrophages reveals that it relies on ~200 transcription factors, ~100 kinases, and ~20 epigenetic enzymes that are differentially expressed in differentiated macrophages compared to their monocytic precursors [reviewed in Ref. (22)].

## Dysregulation in Innate Immune Cells

**Figure 2:**
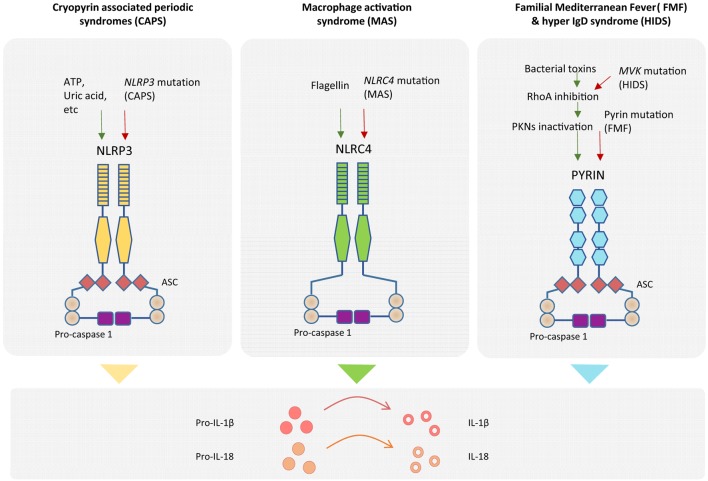
**Genetics of autoinflammatory diseases**. Different inflammasome complexes are activated by different stimulus recognized by specific sensor molecules. Mutations of genes coding inflammasome proteins have been identified in autoinflammatory disorders. Gain-of-function mutations in NLRP3 gene have been detected in cryopyrin-associated periodic syndromes (CAPS), and mutations in NLRC4 gene have been observed in macrophage activation syndrome (MAS). The mechanistic explanation of the exacerbated inflammatory response for patients with Familial Mediterranean Fever (FMF) has recently been described. Mutation of pyrin in FMF patients causes a decrease in pyrin phosphorylation and deregulation of the inflammasome assembly. Higher amounts of inflammasome complex in the different diseases are associated with increased production of mature IL-1β and IL-18 inflammatory cytokines.

Inflammatory responses aiming at destroying invading pathogens consist of very potent effector mechanisms that, if not properly regulated, could potentially be harmful to the host, as illustrated by the appearance of autoinflammatory disorders. In order to provide specificity to the innate immune response, different inflammasomes are defined by the sensor protein that triggers the assembly, such as the NLRP1 that recognizes muramyl dipeptide and anthrax lethal toxin (mouse NLRP1b) (23), NLRP3 that is triggered by several stress-induced molecules including monosodic urate crystals or ATP, NLRC4 that recognizes cytosolic flagellin inflammasomes (24), and the AIM2 inflammasome that assembles in response to cytoplasmic DNA (Figure 2) (25–28). All these cytoplasmic innate immune receptors signal through the adaptor ASC (apoptosis-associated speck-like protein containing a caspase recruitment domain) that recruits caspase-1, leading to the activation of IL-1β and the processing of IL-18 (20). The group of autoinflammatory disorders caused by dysregulation of the inflammasomes is referred to as “inflammasomopathies” (29). Gain-of-function mutations of *NLRP3* leading to aberrant activation of such inflammasomes are the cause of the CAPS spectrum disorders (30). For example, ATP, which is a very well known NLRP3 inflammasome activator and a signal of cellular stress, is released in great amounts by CAPS monocytes when exposed to minute concentrations of inflammatory stimuli that do not induce a reaction in healthy counterparts. CAPS monocytes appear to be more sensitive and prone to generate a stronger reaction to same amounts of LPS compared to monocytes from healthy donors, hence producing an inflammatory feedback loop by secreting large amounts of ATP that will further activate the inflammasome and aberrantly augment the production IL-1β and IL-18 (31). Mutations in *NLRC4* have been shown to cause life-threatening autoinflammatory macrophage activation syndrome with systemic overproduction of IL1-β and IL-18 as well as uncontrolled macrophage activation (32, 33).

In the case of FMF, it was not until this year that a mechanistic explanation of exacerbated IL-1β release by mutated pyrin (coded by *MEFV*) was reported by Park and colleagues. Although pyrin does not seem to bind directly to bacterial products, it is phosphorylated by PKN1 and PKN2 in a RhoA GTPase-dependent manner, leading to inactivation of the pyrin inflammasome formation in the absence of pathogen infection. By contrast, either the presence of several bacterial toxins or the mutation of pyrin in FMF patients results in lack or diminished pyrin phosphorylation, reduced regulation of inflammasome assembly and hyperproduction of cleaved IL-1β (34). Moreover, a molecular link between pyrin (but not NLRP3, AIM2, and NLRC4) inflammasome regulation and the mevalonate kinase pathway has also been recently reported. Mevalonate kinase deficiency generates systemic inflammation with recurrent fever and lymphadenopathy, namely, the hyper IgD syndrome (HIDS) and the more severe mevalonate aciduria. Mevalonate kinase contributes to the inhibition of pyrin expression in an NF-κB-dependent manner through the production of geranyl pyrophosphate, which is necessary for repression of pyrin. As a consequence of absent mevalonate kinase pathways in HIDS patients, *MEFV* is overexpressed, and pyrin is abnormally activated leading to exacerbated inflammatory cytokine release and autoinflammation (35).

## Epigenetics of Autoinflammatory Disorders

Epigenetic is broadly defined as the mitotically heritable changes that affect gene expression without affecting genome DNA sequence. More specifically, epigenetics encompass mechanisms that register, mark or perpetuate gene activity states. It is accepted that, due to their upstream connections with transcription factors and signaling pathways, epigenetic factors sense and mediate interactions between environment (extracellular signals) and the genome. The main epigenetic mechanisms comprise DNA methylation, histone modifications, non-coding RNAs, and chromatin remodeling. DNA methylation occurs by the addition of a methyl group to the 5′ position of a cytosine followed by guanine (CpG dinucleotide). Subsequent demethylation results from the oxidation of 5-methylcytosine catalyzed by ten–eleven translocation enzymes, which forms intermediates (5-hydroxymethylcytosine; 5-formylcytosine; and 5-carboxylcytosine) to yield the final unmethylated cytosines; however, recently, it has been described that these oxidized intermediates may have independent functional roles on their own merit. Posttranslational modifications of different histone amino acid residues are a vast group of epigenetic modifications. The functional role of all these epigenetic modifications depend on various factors including genomic location, and it can be very different in promoters, enhancers, and other genomic sites. Myeloid cells are very plastic, and they display vast changes in epigenetic modifications in response to a variety of environmental stimuli and under pathological inflammatory conditions (36).

In monogenic disorders, such as FMF, studies comparing patients with the same ancestry living in Turkey or in Germany have allowed the determination of the impact of the environment on the severity of FMF, in which environmental factors may contribute to as much as 12% of the phenotypic variation (37). In addition, it has been reported that gains of DNA methylation of the FMF causative gene *MEFV* lead to reduced *MEFV* expression in FMF peripheral leukocytes from 51 FMF patients compared to 21 healthy controls (38).

**Figure 3:**
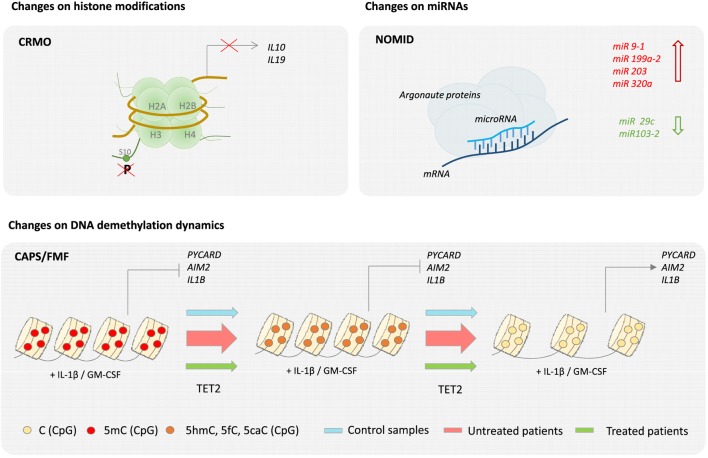
**Epigenetics of autoinflammatory diseases**. Epigenetic changes have been described in several autoinflammatory diseases. For example, in the case of chronic recurrent multifocal osteomyelitis (CRMO), a failure of histone H3 phosphorylation at serine residue 10 (H3S10p) in the promoter region impairs IL-19 and IL-10 expression. Also, neonatal-onset multisystem inflammatory disease (NOMID) patients are associated with an increase of miR 9-1, miR 199a-2, miR 203, and miR 320a and a decrease of miR 29c and miR 103-2 in their skin. Finally, changes on DNA demethylation dynamics have been recently described in cryopyrin-associated periodic syndromes (CAPS) and Familial Mediterranean Fever (FMF).

In the case of other classical monogenic disorders, evidence of epigenetic dysregulation is also starting to emerge from recent studies. Analysis of skin biopsies from NOMID patients, comparing skin lesions with both non-lesional skin and normal skin, is suggestive of epigenetic regulation, as genes that encode histones and enzymes that modify histones were differentially regulated in lesional skin. Moreover, two microRNAs, miR-29c and miR 103-2, were significantly downregulated in lesions, whereas some other skin specific miRNAs including miRNAs miR 9-1, miR 199a-2, miR 203, and miR 320a, were upregulated (39). Nevertheless, a more cell-specific and systematic analysis of the contribution of epigenetics in NOMID pathology is required. Our group has recently described that activation of monocytes and macrophages by inflammatory stimuli, such as cytokines GM-CSF and IL-1β, drives TET2-mediated demethylation of several inflammasome-related molecules including PYCARD, AIM2, IL-1α, and IL-1β. These data led us to further investigate the methylation status of inflammasome genes in a cohort of CAPS and FMF patients. We found that demethylation of such genes is exacerbated in untreated CAPS patients and that this demethylation was reverted by anti-IL-1β treatment (40). We provided evidence for the first time that an epigenetic mechanism, in this case DNA methylation, may contribute the decrease in IL-1β production threshold in CAPS patients, and provide the basis for the discovery of novel biomarkers that could complement the diagnosis of autoinflammatory disorders (Figure 3). Evidence for epigenetic dysregulation has also been provided in the case of complex autoinflammatory disorders such as Behçet’s disease. Genome-wide DNA methylation studies in monocytes and CD4+ cells of BD patients, during flares and remission, comparing to healthy counterparts have revealed significant differences in methylation levels throughout the genome. Moreover, BD monocytes displayed 383 differentially methylated CpGs in 228 genes, whereas CD4+ showed 125 differential CpGs in 62 genes. Both hypermethylation and hypomethylation were represented in equivalent levels, and GO analysis of affected genes revealed an overrepresentation of cytoskeletal remodeling genes in monocytes and antigen processing and antigen presentation in CD4+ lymphocytes. Interestingly, BD patients in remission showed similar DNA methylation patterns as healthy controls, suggesting that changes in global DNA methylation patterns directly reflect disease pathology (41). In the case of CRMO, an autoinflammatory disease affecting the bone, an imbalance of proinflammatory and regulatory signals has been described. In particular, decreased expression of *IL10* has been shown to be directly attributed to epigenetic dysregulation. CRMO monocytes fail to produce IL-10, and related anti-inflammatory cytokine IL-19, upon LPS stimulation, which in turn leads to IL-1β overproduction and inflammation within the bone. *IL10* repression is suggested to occur through impaired chromatin remodeling caused by altered histone H3 phosphorylation at serine residue 10 at the *IL10* proximal promoter, which also encompasses the regulatory elements of the *IL19* (*CNS1*) gene and partially the *IL20 gene* (*CNS2*). In addition, differential DNA methylation of the *IL10* intronic enhancer element (I-SRE) and the *IL19* CNS1 was also observed. This strongly suggests that epigenetic regulation contribute to the overall proinflammatory imbalance and pathophysiology in CRMO (42, 43). Another set of multifactorial, complex disorders are the group of IBDs, typically CD and ulcerative colitis (UC), in which genetic predisposition, environmental microbiota, and immune responses are the main contributing factors to its pathology. Regarding to genetic contribution, twin studies show a 50% concordance for monozygotic and 10% for dizygotic twins for disease development (16). Using methylation bead arrays to compare whole blood from 21 CD adults versus 19 sex-matched controls, as well as 16 CD pediatric patients, a specific methylation profile for CD was determined, which includes differential methylation in several immune related genes such as *MAPK13, FASLG, PRF1, S100A13, RIPK3*, and *IL21R* in patients compared to healthy controls (44). Interestingly, the DNA methyltransferase gene *DNMT3A* has been identified by GWAS as a CD susceptibility gene, which suggests that aberrant DNA methylation may be participating in CD etiology (45). Although specific profiles of miRNA expression in UC and CD have been described in both target tissues and blood, cell type-specific miRNA expression data to unambiguously assess causality are still lacking (46).

Altogether, epigenetic dysregulation is emerging as a relevant contributing factor of autoinflammatory development, and further investigation would provide valuable insight into their pathogenesis that could hint for molecular-tailored treatment.

## Conclusion

The possibility of additional causative mechanisms leading to exacerbated autoinflammation in both mutated and non-mutated pathogenic genes increases the complexity of how autoinflammatory diseases manifest and evolve. It is conceivable that different gene variants could behave in a differential manner depending on its association with non-genetic background, which in turn is able to shape disease presence and severity, ranging from being a true causative mutation, a functional polymorphisms or remaining silent (47). In this respect, in addition to more in-depth genetic studies using massively parallel sequencing techniques (such as whole-exome sequencing and targeted deep resequencing), epigenetic genome-wide profiling studies could be of great value as they would inform of non-genetic landscapes that contribute to pathogenicity. Moreover, current genetic diagnosis of a few candidate genes would expand potential biomarkers taking into account clinical and molecular traits other than described mutations. Overall, the identification of epigenetic dysregulation contributing to autoinflammation will allow us to address environmental contribution to autoinflammatory syndromes.

## Author Contributions

DÁ-E and EB wrote the manuscript. RV-T wrote the manuscript and made figures.

## Conflict of Interest Statement

The authors declare that the research was conducted in the absence of any commercial or financial relationships that could be construed as a potential conflict of interest.
